# Memories and post-traumatic stress-related symptoms in older, post-cardiac surgery patients: substudy of an RCT

**DOI:** 10.1186/cc10949

**Published:** 2012-03-20

**Authors:** N Hammond, F Bass, Y Shehabi

**Affiliations:** 1 Prince of Wales Hospital, Randwick, Australia

## Introduction

The majority of ICU survivors display little evidence of severe psychological sequelae. However, there is evidence of post-traumatic stress disorder (PTSD)-related symptoms such as anxiety, depression, panic attacks, distressing memories and flashbacks within the first 3 months post ICU discharge [1,2]. This substudy of the DEXCOM trial 3 (randomised controlled trial of neurobehavioural effects of dexmedetomidine or morphine for sedation and analgesia in patients 60 years or older, undergoing coronary artery bypass grafting and/or valve replacement) aims to explore any negative memories of the ICU and development of PTSD-related symptoms between treatment groups of patients at high risk of developing delirium.

## Methods

At 8 weeks post ICU discharge, patients completed three assessment tools, by mail or telephone. Tools used were Depression Anxiety Stress Scale, ICU memory assessment tool and impact of events scale.

## Results

A total of 153 patients completed the substudy; 72 patients in the [M]orphine group and 81 in the [D]exmedetomidine group. The mean age (years) in the M group was 72 (SD 5) and in the D group 69 (SD 6), with 71% (*n *= 51) males in the M group and 84% (*n *= 68) in the D group. The mean ICU hours for M and D were 58 (SD 40) and 48 (SD 32) respectively. No significant differences of memories or PTSD-related symptoms between the two treatment groups, for each of the three assessment tools, were found. From the ICU memory tool, 21% (*n *= 15/70) of M group patients and 15% (*n *= 12/81) of the D patients remember being in the ICU. Just over one-half of the patients in both groups did not remember all of their ICU stay with clarity (M group: 54%, *n *= 39/72; D group: 51%, *n *= 40/78). Furthermore, 23% (*n *= 15/64) of M patients and 14% (*n *= 10/73) of D patients had intrusive memories whilst in the ICU.

## Conclusion

Patients undergoing cardiac surgery with ICU stay do not have clear memories of this episode. A small number had intrusive memories, which are more common in M patients. The study used a convenience sample so was not powered to detect a significant difference. No differences in factual or delusional memories or PTSD-related symptoms between the treatment groups were found. These data could be the basis of a sample size calculation for a larger study.
